# Meta-analysis of the effect of the pringle maneuver on long-term oncological outcomes following liver resection

**DOI:** 10.1038/s41598-021-82291-4

**Published:** 2021-02-08

**Authors:** Elias Khajeh, Saeed Shafiei, Sadeq Ali-Hasan Al-Saegh, Ali Ramouz, Ahmed Hammad, Omid Ghamarnejad, Mohammed Al-Saeedi, Nuh Rahbari, Christoph Reissfelder, Arianeb Mehrabi, Pascal Probst, Hani Oweira

**Affiliations:** 1 Division of Liver Surgery and Visceral Transplantation, Department of General, Visceral, and Transplantation Surgery, University of Heidelberg, Im Neuenheimer Feld 420, 69120 Heidelberg, Germany; 2 Liver Cancer Center Heidelberg (LCCH), Heidelberg, Germany; 3 Department of Surgery, Universitätsmedizin Mannheim, Medical Faculty Mannheim, Heidelberg University, Mannheim, Germany

**Keywords:** Diseases, Medical research

## Abstract

Hepatic pedicle clamping reduces intraoperative blood loss and the need for transfusion, but its long-term effect on survival and recurrence remains controversial. The aim of this meta-analysis was to evaluate the effect of the Pringle maneuver (PM) on long-term oncological outcomes in patients with primary or metastatic liver malignancies who underwent liver resection. Literature was searched in the Cochrane Central Register of Controlled Trials (CENTRAL), Medline (via PubMed), and Web of Science databases. Survival was measured as the survival rate or as a continuous endpoint. Pooled estimates were represented as odds ratios (ORs) using the Mantel–Haenszel test with a random-effects model. The literature search retrieved 435 studies. One RCT and 18 NRS, including 7480 patients who underwent liver resection with the PM (4309 cases) or without the PM (3171 cases) were included. The PM did not decrease the 1-year overall survival rate (OR 0.86; 95% CI 0.67–1.09; P = 0.22) or the 3- and 5-year overall survival rates. The PM did not decrease the 1-year recurrence-free survival rate (OR 1.06; 95% CI 0.75–1.50; P = 0.75) or the 3- and 5-year recurrence-free survival rates. There is no evidence that the Pringle maneuver has a negative effect on recurrence-free or overall survival rates.

## Introduction

Liver resection remains the only curative treatment for hepatic malignancies, and can improve long-term survival^1^. Improvements in surgical techniques, better selection of patients, and improved perioperative care have increased the number of hepatectomies performed worldwide each year^1,2^. There is growing evidence that excessive blood loss during hepatectomy and the subsequent need for blood transfusions may contribute to a poor outcome for non-cirrhotic and cirrhotic liver resections^1,2^. Perioperative blood transfusion has been associated with recurrence and poorer long-term survival due to an immune response dysfunction^3^.

Vascular occlusion techniques have been used by some surgeons during hepatic resection to minimize intraoperative blood loss, especially in large tumors or tumors that are adjacent to major vessels^4,5^. Pringle described a technique whereby transient hepatic inflow was occluded by clamping the portal triad. Portal clamping in the Pringle maneuver (PM) has been modified several times in form of intermittent portal clamping^6,7^ and selective portal clamping^8^. These modifications can control intraoperative blood loss and decrease the need for transfusion. Some surgeons believe that this reduction in the rate of blood transfusions can improve long-term oncological outcomes. On the other hand, some argue that the PM may increase the risk of ischemia–reperfusion injury to the liver, which may impair hepatocyte function^4,6,7^.

The present systematic review and meta-analysis aimed to evaluate the effect of the PM on long-term oncological outcomes in patients with primary or metastatic liver malignancies who underwent liver resection.

## Results

### Literature search strategy and included studies

**Figure 1:**
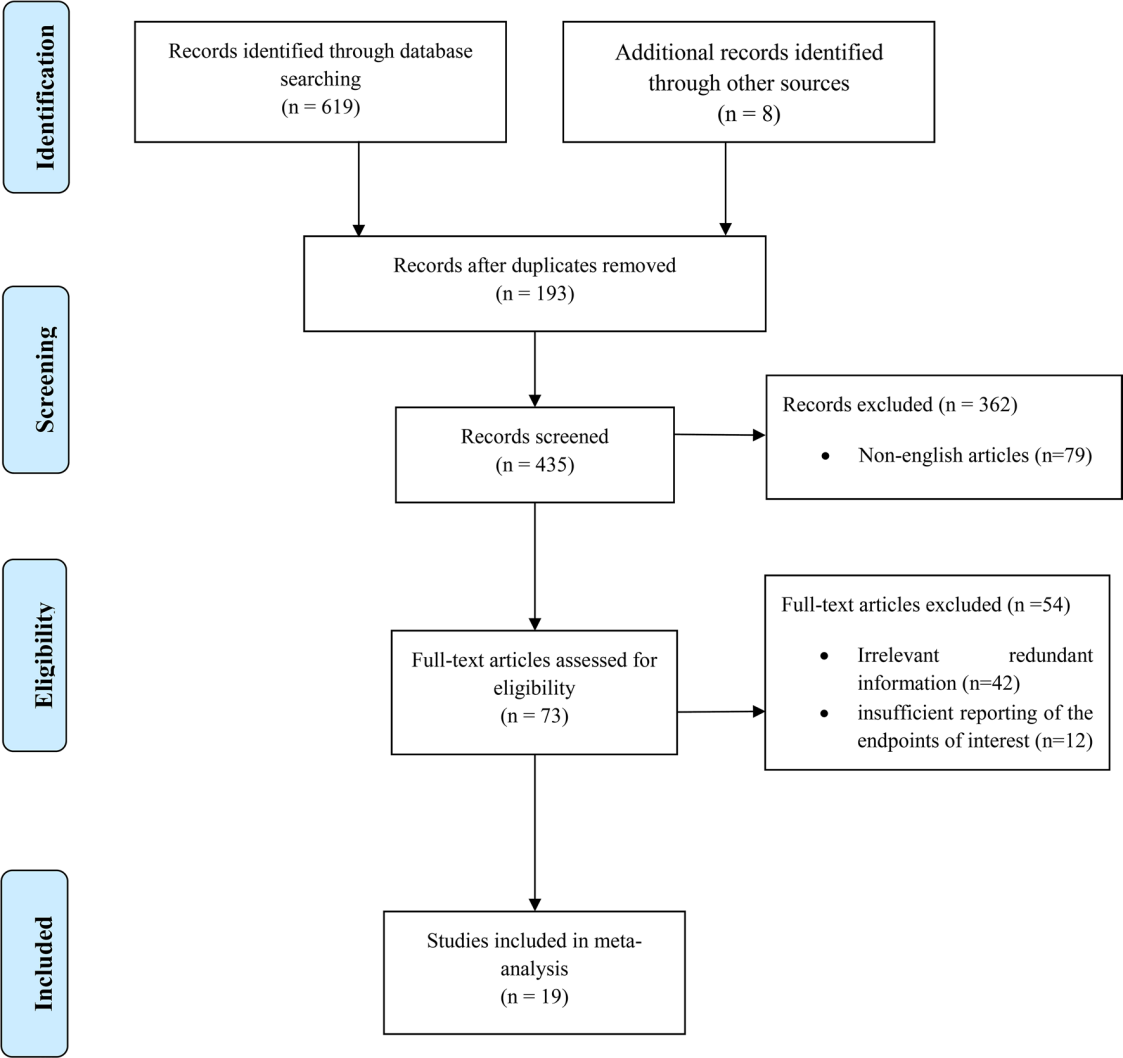
PRISMA flow chart of study selection.

**Figure 2:**
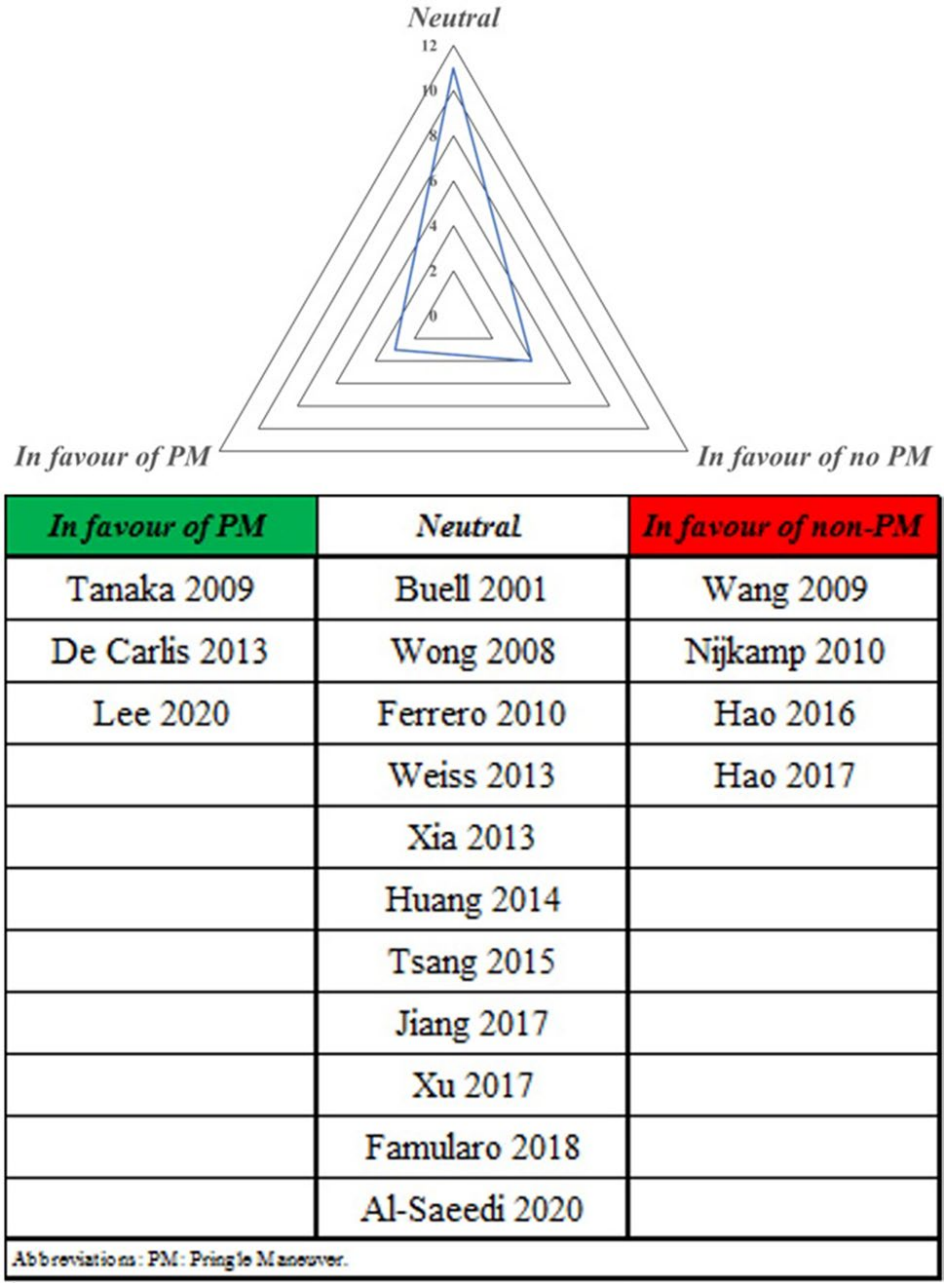
Distribution of studies according to oncological outcomes of Pringle maneuver.

The literature search retrieved 435 studies excluding duplicates. Of these, 416 papers were excluded for various reasons, including redundant information and insufficient data on survival. In the end, 19 articles were included in the current meta-analysis (Fig. 1). During the primary evaluation, included articles were subdivided into three groups regarding their suggestions and conclusion on the effect of PM on oncological outcomes of the patients: in favor of the PM, neutral, and not in favor of the PM (Fig. 2).

### Risk of bias assessment for included studies

Of the 19 articles included in this meta-analysis, only one was an RCT. This study included 80 patients (39 cases with PM and 41 cases without PM). The other 18 NRS included 7400 patients (4270 cases with PM and 3130 cases without PM). All studies were published between 2002 and 2020 (Table 1). As shown in Table 2, most studies had moderate bias.

**Table 1:** Characteristics of included studies.

Author (year)	Country	Study type	Age		Sample size		Type of hepatectomy		Duration of Pringle (min)	Type of Pringle	Diagnosis
			PM	NPM	PM	NPM	PM	NPM			
Al-Saeedi (2020)^9^	Germany	Retrospective cohort	58.4	60.5	50	159	All patients underwent extended hepatectomy		19	Intermittent	HCC and CRLM
Lee (2019)^10^	China	Retrospective cohort	58	60.5	88	88	Minor: 51 (58.0%) Major: 37 (42.0%)	Minor: 54 (61.4%) Major: 34 (38.6%)	Mean (range) 45 (15–87)	Intermittent	HCC
Famularo (2018)^4^	Italy	Retrospective cohort	65.1	67.6	176	265	Minor: 153 (87.4%) Major: 22 (12.6%)	Minor: 228 (86.4%) Major: 36 (13.6%)	Mean (range) 23 (14–30)	Intermittent	HCC
Jiang (2017)^11^	China	Retrospective cohort	NA	NA	132	112	NA	NA	NA	Intermittent	HCC
Xu (2017)^12^	China	Retrospective cohort	56.02	56.10	290	296	Minor: 38 (13.10%) Major: 105 (36.20%)	Minor: 94 (31.75%) Major: 126 (42.57%)	163 cases < 15,127 cases 15–30	Continuous	HCC
Hao (2017)^6^	China	Retrospective cohort	52.65	55	303	52	Minor: 122 (40%) Major: 181 (60%)	Minor: 25 (48%) Major: 27 (52%)	NA	Intermittent	HCC
Hao (2016)^13^	China	Retrospective cohort	52.65	55	206	60	Minor: 79 (38.3%) Major: 127 (61.6%)	Minor: 25 (41.6%) Major: 35 (58.3%)	29.6	Intermittent	HCC
Tsang (2015)^14^	Canada	Retrospective cohort	63.0	63.0	110	110	Minor: 41 (37.2%) Major: 69 (63.3%)	Minor: 43 (39%) Major: 67 (60.9%)	Mean (range) 20 (15–30)	Intermittent	CRLM
Huang (2014)^15^	China	Retrospective cohort	56.65	54.2	931	618	Minor: 592 (63.4%) Major: 416 (44.6%)	Minor: 326 (52.7%) Major: 289 (46.7%)	Mean (range) 47.4 (3–208)	Intermittent	HCC
Weiss (2013)^16^	USA	Retrospective cohort	62.7	64.3	874	54	Minor: 286 (32.7%) Major: 548 (66.8%)	Minor: 15 (27.7%) Major: 39 (72.2%)	Mean (range) 35 (1–181)	prolonged PM(> 60 min) and short (< 60 min)	CRLM
Xia (2013)^17^	China	Prospective cohort	48	57	224	162	Minor: 131 (58.4%) Major: 93 (41.5%)	Minor: 85 (52.4%) Major: 77 (47.5%)	Mean (range) 50 (30–98)	Intermittent	HCC
De Carlis (2013)^18^	Italy	Case-matched	61	61	60	60	Minor: 36 (60%) Major: 24 (40%)	Minor: 34 (56.6%) Major: 26 (43.3%)	NA	Intermittent	CRLM
Ferrero (2010)^19^	Italy	Randomized-controlled	61.3	64.8	39	41	Minor: 19 (48.7%) Major: 20 (51.2%)	Minor: 22 (53.6%) Major: 19 (46.3%)	Mean (SD) 47.8 (17.2)	Intermittent	CRLM
Nijkamp (2010)^20^	Netherlands	Retrospective cohort	NA	NA	50	72	All patients underwent partial hepatectomy		21 (2–69) and 40 (20–90)	Intermittent, continuous	CRLM
Giuliante (2010)^21^	Italy	Retrospective cohort	62 ± 10		188	355	228 cases (42%) underwent major hepatectomy 315 cases ( 58%) underwent minor hepatectomy		NA	Intermittent, continuous	CRLM
Wang (2009)^22^	Taiwan	Retrospective cohort	NA	NA	114	359	NA	NA	NA	Intermittent	HCC
Wong (2008)^23^	UK	Retrospective cohort	NA	NA	289	274	Minor: 19 (48.7%) Major: 150 (51.9%)	Minor: 22 (53.6%) Major: 143 (52.18%)	Mean (range) 22 (2–104)	Intermittent	CRLM
Tanaka (2008)^24^	Japan	Retrospective cohort	NA	NA	100	19	NA	NA	NA	Intermittent	HCC
Buell (2002)^25^	USA	Retrospective cohort	58	62.3	85	15	NA	NA	NA	Intermittent	CRLM

**Table 2:** Assessment of study quality.

ROBINS-I tool								
Author (year)	Confounding	Participant selection	Classification of intervention	Deviation from intended intervention	Missing data	Outcome measurement	Selection of reported results	Overall bias
Al-Saeedi (2020)^9^	Low	Low	Low	No information	Low	Low	No information	Moderate
Lee (2019)^10^	Low	Low	Low	Low	Low	Low	No information	Low
Famularo (2018)^4^	Low	No information	Low	No information	Low	Low	No information	Moderate
Jiang (2017)^11^	Low	Low	Low	No information	Low	Low	No information	Moderate
Xu (2017)^12^	Low	No information	Low	No information	Low	Low	No information	No information
Hao (2017)^6^	Low	Low	Low	No information	Low	Low	No information	Moderate
Hao (2016)^13^	Low	Low	Low	No information	Low	Low	No information	Moderate
Tsang (2015)^14^	Low	Low	Low	Low	Low	Low	No information	Low
Huang (2014)^15^	Low	Low	Low	No information	Low	Low	No information	Moderate
Weiss (2013)^16^	Low	Low	Low	No information	Low	Low	No information	Moderate
Xia (2013)^17^	Low	Low	Low	No information	Low	Low	No information	Moderate
De Carlis (2013)^18^	Low	Low	Low	No information	Low	Low	No information	Moderate
Nijkamp (2010)^20^	No information	Low	Low	No information	Moderate	Low	No information	Moderate
Giuliante (2010)^21^	low	Low	Low	No information	Moderate	Low	No information	Moderate
Wang (2009)^22^	Low	No information	Low	No information	Low	Low	No information	No information
Wong (2008)^23^	Low	No information	Low	No information	Low	Low	No information	No information
Tanaka (2008)^24^	Low	No information	Low	Low	Low	Low	No information	Moderate
Buell (2002)^25^	Moderate	No information	Low	No information	Low	Low	No information	Moderate

Cochrane risk of bias tool for randomized controlled trials								
First author	Ferrero (2010)^22^							
Bias arising from the randomization process	Some concerns							
Bias arising from the timing of identification and recruitment of individual participants in relation to timing of randomization	Some concerns							
Bias due to deviations from intended interventions	Some concerns							
Bias due to missing outcome data	Low risk							
Bias in measurement of the outcome	Some concerns							
Bias in selection of the reported result	Some concerns							
Overall bias	Some concerns							

### Recurrence-free survival rate

#### One-year recurrence-free survival rate

**Figure 3:**
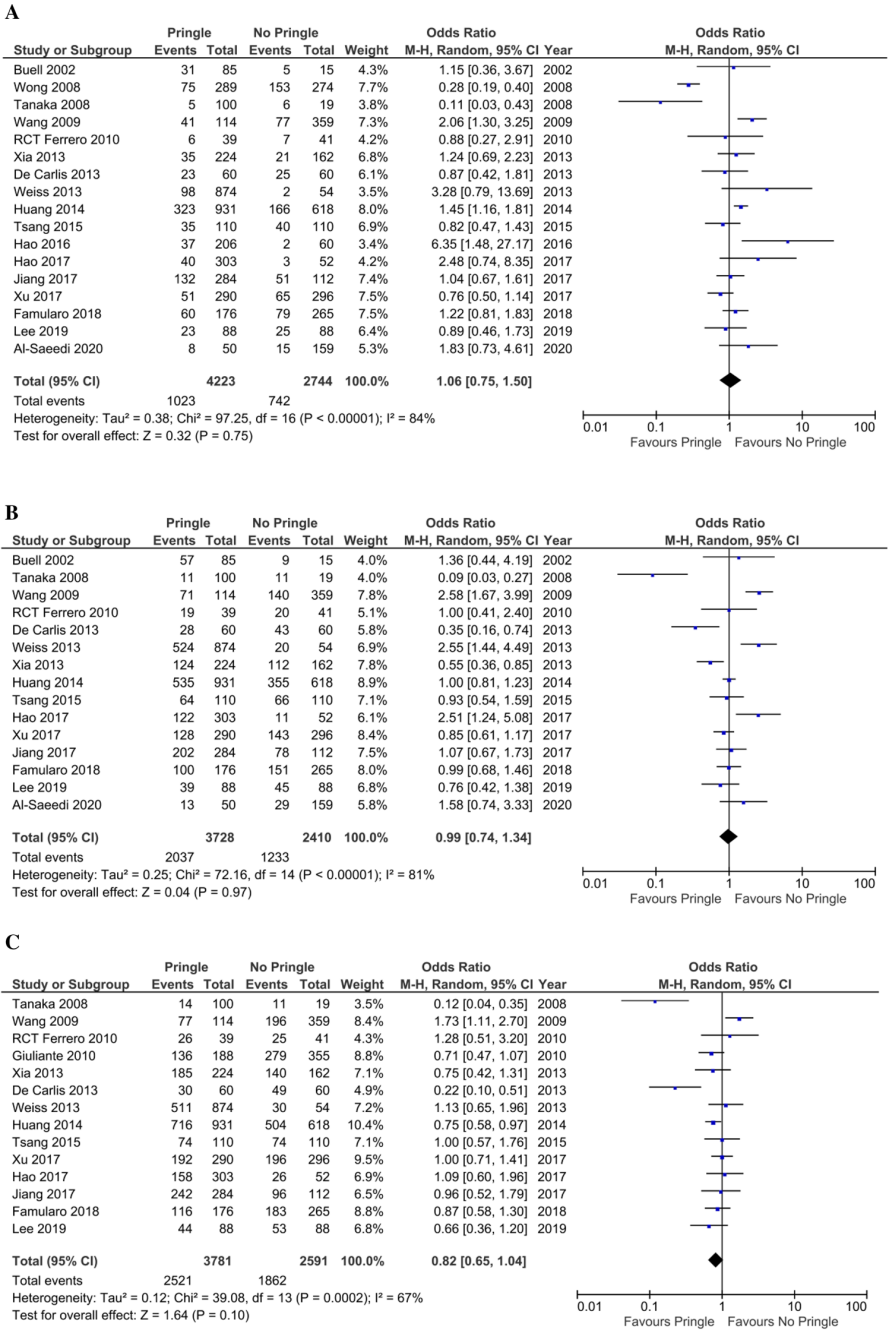
(**A**) Forest plot showing 1-year recurrence of hepatic malignant lesions. (**B**) Forest plot showing 3-year recurrence of hepatic malignant lesions after hepatectomy. (**C**) Forest plot showing 5-year recurrence of hepatic malignant lesions after hepatectomy.

One-year RFS rates were reported for 6758 patients from 17 studies (4223 patients were in the PM group and 2744 patients in the non-PM group). The recurrence of malignant hepatic lesions was reported in 1023 cases (24.2%) in the PM group and in 742 cases (27%) in the non-PM group. Meta-analysis indicated that the PM did not decrease 1-year RFS rate (OR 1.06; 95% CI 0.75–1.50; P = 0.75; Fig. 3A) using a random-effects model. There was considerable heterogeneity among the studies (I^2^ = 84%; P < 0.00001).

#### Three-year recurrence-free survival rate

Recurrence of malignant lesions during the first 3 years after hepatectomy was reported in 6138 cases from 15 studies. Of these, recurrence was reported in 2037 patients (54.6%) in the PM group and in 1233 patients (51.1%) in the non-PM group. Meta-analysis revealed no significant difference in 3-year RFS rate between the groups (OR 0.99; 95% CI 0.74–1.34; P = 0.97) using the random-effects model (Fig. 3B). The studies that reported 3-year RFS rates were not homogeneous (I^2^ = 81%; P < 0.00001).

#### Five-year recurrence-free survival rate

A total of 14 studies with 3781 patients in the PM group and 2591 patients in the non-PM group reported 5-year recurrence. As is seen in Fig. 3C, recurrence was reported in 2521 patients (66.67%) in the PM group and in 1862 patients (71.86%) in the non-PM group. The meta-analysis showed that 5-year RFS rate is not significantly different between the two groups (OR 0.82; 95% CI 0.65–1.04; P = 0.1) using the random-effects model (Fig. 3C). The studies that reported 5-year RFS rates were not homogeneous (I^2^ = 67%; P = 0.0002).

### Overall survival rates

#### One-year overall survival rate

**Figure 4:**
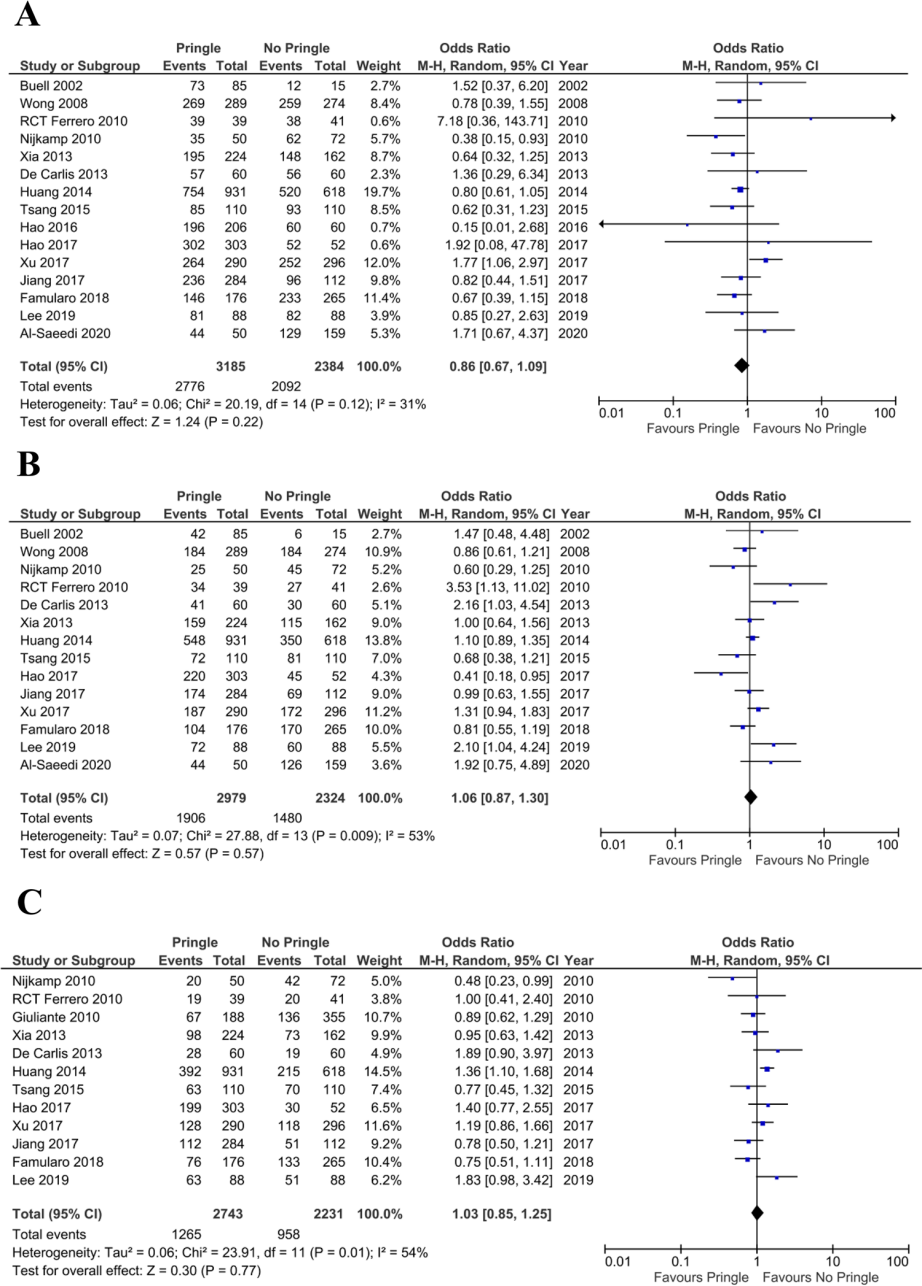
(**A**) Forest plot showing 1-year survival of patients with hepatic malignant lesions. (**B**) Forest plot showing 3-year survival of patients with hepatic malignant lesions. (**C**) Forest plot showing 5-year survival of patients with hepatic malignant lesions.

Fifteen studies including 5569 patients reported the 1-year OS rate. Of these, 2776 patients (87.15%) were in the PM group and 2092 patients (87.7%) were in the non-PM group. According to our analysis using the random-effects model, the 1-year OS rate was not significantly different between the PM and non-PM group (OR 0.86; 95% CI 0.67–1.09; P = 0.22) (Fig. 4A). The I^2^ was 31% with a P value of 0.12.

#### Three- and five-year overall survival rates

The 3-year OS rate was 64% in the PM group and 63.6% in the non-PM group. The 5-year OS rate was 46.11% in the PM group and 42.9% in the non-PM group (Fig. 4B,C). Meta-analysis indicated that the 3- and 5-year OS rates were not significantly different between the PM and non-PM groups.

### Subgroup analysis

The type of malignant tumor (i.e., primary or metastatic) had no significant effect on the 1-year RFS rate in the PM and non-PM groups (primary tumors: OR 1.16; 95% CI 0.86–1.56; P = 0.34; metastatic tumors: OR 0.82; 95% CI 0.43–1.56; P = 0.56), nor did it have an effect on the 3-year RFS rate (primary tumors: OR 0.96; 95% CI 0.68–1.37; P = 0.84; metastatic tumors: OR 1.10; 95% CI 0.60–2.02; P = 0.77) or the 5-year RFS rate (primary tumors: OR 0.84; 95% CI 0.63–1.12; P = 0.24; metastatic tumors: OR 0.77; 95% CI 0.47–1.24; P = 0.28).

The type of malignant tumor had no significant effect on the 1-year OS rate in the PM and non-PM groups (primary tumors: OR 0.87; 95% CI 0.67–1.14; P = 0.31; metastatic tumors: OR 0.74; 95% CI 0.49–1.12; P = 0.15), nor did it have an effect on the 3-year OS rate (primary tumors: OR 1.04; 95% CI 0.85–1.29; P = 0.69; metastatic tumors: OR 1.12; 95% CI 0.72–1.76; P = 0.61) or 5-year OS rate (primary tumors: OR 1.10; 95% CI 0.88–1.38; P = 0.39; metastatic tumors: OR 0.89; 95% CI 0.62–1.28; P = 0.53) (Supplemental Figs. 1 and 2).

## Discussion

Intraoperative bleeding is one of the most common and life-threatening complications during liver surgery, and has been associated with increased long-term morbidity and mortality^26^. In addition, intraoperative hemorrhage increases the rate of blood transfusions, which have a negative impact on long-term postoperative outcomes by reducing the patient’s immune defense^26,27^. Excessive bleeding and blood transfusion also reduce patient survival^26,27^. Excessive intraoperative bleeding and vascular occlusion are both associated with an increased risk of postoperative surgical complications and unfavorable clinical outcomes. Therefore, the optimal approach to liver resection is to perform surgery without hepatic vascular occlusion while minimizing blood loss and the need for blood transfusion.

Despite several strategies to reduce intraoperative bleeding, the PM remains the most commonly used technique because it was shown to reduce blood loss with high efficacy in initial randomized trials^10,26^. However, some studies have not confirmed these initial findings and have even suggested a higher risk of ischemia–reperfusion injury for healthy liver tissue^28,29^. Furthermore, an increased rate of postoperative complications has been shown in patients who undergo PM during hepatectomies in some studies^30^. To prevent liver injuries, portal pedicle clamping was modified in the PM to an intermittent approach^31^. Despite this modification, the overall efficacy of the PM remains controversial^32,33^. Whether the PM promotes liver injury remains a topic of debate. Furthermore, how the PM affects recurrence and survival in patients with malignant lesions who underwent hepatectomy is not well understood. Although some studies have suggested that prolonged PM increases recurrence^1,20^, others have demonstrated no effect^15,19,34^. For instance, Al-Saeedi et al. revealed that a PM of less than 20 min did not increase the recurrence rate after 3 years^9^. Recent studies showed that the PM has no significant positive impacts on clinical outcomes after minor liver surgeries^13,32^. However, major liver resections, which have more intraoperative blood loss, probably benefit more from the PM. To address this controversy, we performed a meta-analysis to compare the long-term oncological outcomes of hepatectomy with and without a PM.

The PM, regardless of whether it is complete or intermittent, was shown to be an independent risk factor for cancer recurrence in one study^13^. However, other studies have reported no negative impact of the PM on patient survival and disease recurrence^17,18^. In a recent randomized-controlled trial, the intermittent PM did not affect disease-free survival after hepatectomy, but did improve the OS rate^10^. The positive effect of the intermittent PM was particularly promising in patients with hepatic disorders such as cirrhosis^10^. In the present analysis, we observed no significant differences in 1-, 3-, and 5-year overall and recurrence-free survival between the PM and non-PM groups. Furthermore, subgroup analysis revealed no significant effects of tumor type (i.e., primary or metastatic) on 1-, 3-, and 5-year survival between the PM and non-PM groups. This is in accordance with previous findings from large patient cohorts and clinical trials.

The PM was shown to be a risk factor for disease recurrence in several studies. It has been hypothesized that ischemia during portal pedicle clamping causes microvascular damage by breaking adhesions between tumor cells and endothelial cells^35^. The hepatic ischemia-perfusion cycle might increase the expression of E-selectin, which plays a crucial role in cancer cell metastasis^36,37^. However, we found no significant increase in disease recurrence following hepaectomy with the PM, indicating that the PM is not associated with disease recurrence after hepatectomy.

During reperfusion, liver parenchymal cells are thought to be injured by cytokines and radical oxygen species, which are produced by active Kupffer cells^38^. However, a meta-analysis reported no significant patient benefits of hemihepatic vascular occlusion over complete hepatic vascular occlusion, despite a lower rate of liver injury^39^. This suggests that significant hepatic injury is not caused by the PM, and that the potential benefits outweigh the potential disadvantages. In addition, of enrolled studies in this meta-analysis, four studies (2335 cases) reported the number of patients with steatosis, and no significant difference was observed in means of fatty liver distribution among patients with and without PM a. However, included studies failed to provide more detailed data on clinical or oncological impacts of liver texture characteristics (e.g. macrovesicular or microvesicular liver steatosis, or liver fibrosis) on outcomes of the pringle maneuver, which prohibited us from carrying out subgroup analyses.

A study by Fagenson et al. reported that patients undergoing minor liver resection and cases with metastatic disease had a worse outcome when PM was performed^40^. This finding is in similar line with our previously published report. Our results showed that PM is useful in patients who underwent extended liver resection, but this surgical maneuver may not be beneficial in minor hepatectomies^9^. It can be derived that PM is associated with encouraging early perioperative outcomes without worsening the long-term survival among well-selected patients. On this basis, it cannot be denied that the selection of patients undergoing PM plays a principal role in increasing of safety and efficacy of PM.

There are some limitations to the present study. The main weakness is the variability in PM techniques, underlying liver disease, tumor stage status, and preoperative liver function between the included studies. Due to lack of subgroup results regarding the underlying liver disease, especially liver cirrhosis, it was not possible to assess the impact of PM in cirrhotic patients. In addition, although several studies have compared the PM with non-PM techniques, the number of RCTs is low, and most studies have a retrospective design, which can have a selection bias because PM enable surgeons to perform more aggressive hepatectomy in patients with more advance tumors with worse prognosis. We have added to study from the same center in our meta-analysis^6,13^; the first study was performed between January 2007 and December 2010^13^ and the second study was performed between January 2010 and December 2012^6^. These two studies may include overlapping patients in 2010 which can create some bias in present meta-analysis.

In conclusion, the present study shows that the PM is a suitable surgical technique for managing intraoperative bleeding during liver resection, and does not increase tumor recurrence and long-term mortality. We believe that the PM is a useful and acceptable aopproach to major or extended liver resection. However, further studies in large patient cohorts and randomized trials are needed to comprehensively evaluate the advantages and disadvantages of this procedure.

## Methods

This systematic review and meta-analysis was reported according the Preferred Reporting Items for Systematic Reviews (PRISMA) guidelines^41^.

### Eligibility criteria

The research question was formulated according to the PICOS strategy.

- Population: all adult patients who underwent liver resection
- Intervention: PM during liver resection
- Comparators: no PM
- Outcome: overall or recurrence-free survival rates
- Study design: all study types methodological designs, including human subjects, except case series with less than ten patients, narrative or systematic reviews, letters, conference abstracts, and study protocols.

Duplicate publications or overlapping cohorts were excluded.

### Search strategy

According to Goossen et al.^42^ the following databases were searched.

1. Cochrane Central Register of Controlled Trials (CENTRAL)
2. Medline (via PubMed)
3. Web of Science

Databases were last searched for relevant publications in May 2020. The references of each included study were also searched for additional relevant articles. The combination of search terms is presented in Supplemental Text 1.

### Study selection

Two investigators (SS and AH) independently screened all papers identified by the search strategy and selected eligible studies based on the PICOS criteria. Two authors (SAHS and AR) then reviewed and evaluated the full-text of eligible articles and extracted the data. Discrepancies were settled by a discussion with a third author (EK).

### Outcomes and data items

#### Recurrence-free survival rate

The recurrence-free survival (RFS) rate was defined as the number of the patients who survived without signs of recurrence after primary liver resection. We measured the RFS after 1, 3, and 5 years.

#### Overall survival rate

The overall survival (OS) rate was defined as the number of patients who survived after liver resection, regardless of disease recurrence. We measured the OS at 1, 3, and 5 years.

### Quality assessment

The Cochrane risk-of-bias tool was used to assess the quality of randomized-controlled trials (RCT) and the ROBINS-I tool was used to assess the quality of non-randomized studies (NRS)^43,44^. The Cochrane risk-of-bias tool evaluated several items, including bias arising from the randomization process, bias arising from the timing of identification and recruitment of individual participants in relation to the timing of randomization, bias due to deviations from intended interventions, bias due to missing outcome data, and bias in the selection of the reported result. The overall risk of bias was low if the study was judged to be at low risk of bias for all domains. There were some concerns of bias if some concern of bias was detected in at least one domain. The risk of bias was high if the study was judged to be at high risk of bias in at least one domain or if some concerns of bias were detected in multiple domains.

### Statistical analysis

Statistical analyses were performed by RevMan version 5.3 (Nordic Cochrane Centre, Cochrane Collaboration, Copenhagen, Denmark). Pooled results were analyzed using the Mantel–Haenszel method. Results were presented as odds ratios (OR) or as survival rates with 95% confidence intervals (CI). Because of clinical heterogeneity between studies, a random‐effects model was used. A P value < 0.05 for the Q-test or a *I*^2^ index more than 75% indicated statistical heterogeneity among studies. An *I*^*2*^ index between 50 and 75% indicated moderate statistical heterogeneity.

## Supplementary Information

Supplementary Information 1.

Supplementary Information 2.

Supplementary Information 3.

Supplementary Information 4.

Supplementary Information 5.

Supplementary Information 6.

Supplementary Information 7.
