# Supplementary material for: Meta-analysis of the effect of the pringle maneuver on long-term oncological outcomes following liver resection
Source: Sci Rep. 2021 Feb 8;11:3279. doi: 10.1038/s41598-021-82291-4 (PMC7870962; doi:10.1038/s41598-021-82291-4)
Supplement: Supplementary file 1 — Supplementary Information 1. [file 41598_2021_82291_MOESM1_ESM.docx]

**Meta-analysis of the Effect of the Pringle Maneuver on Long-Term Oncological Outcomes Following Liver Resection**

Elias Khajeh^1^, Saeed Shafiei^1^, Sadeq Ali-Hasan Al-Saegh^1^, Ali Ramouz^1^, Ahmed Hammad^1^, Omid Ghamarnejad^1^, Mohammed Al-Saeedi^1^, Nuh Rahbari^3^, Christoph Reissfelder^3^, Arianeb Mehrabi ^1,2^, Pascal Probst^1^, Hani Oweira^3^

^1^ Department of General, Visceral, and Transplantation Surgery, University of Heidelberg, Heidelberg, Germany;

^2^ Liver Cancer Center Heidelberg (LCCH), Heidelberg, Germany

^3^Department of Surgery, Universitätsmedizin Mannheim, Medical Faculty Mannheim, Heidelberg University, Mannheim, Germany.

**Short title:** Oncological outcomes following Pringle maneuver in hepatectomy

**Correspondence to:**

Prof. Dr. med. A. Mehrabi, FICS, FEBS, FACS

Head of the Division of Liver Surgery and Visceral Transplantation

Department of General, Visceral, and Transplantation Surgery

University of Heidelberg, Im Neuenheimer Feld 110, 69120 Heidelberg, Germany

Tel: 0049 – 6221 – 5636223; Fax: 0049 – 6221 - 567470

E-Mail: arianeb.mehrabi@med.uni-heidelberg.de

**Sources of funding:** None

**Type of article:** Meta-analysis

**Supplemental Text-1:** The combination of search terms

(“Pringle” OR “inflow occlusion” OR “inflow clamping” OR “inflow exclusion” OR “flow occlusion” OR “flow clamping” OR “flow exclusion” OR “Vascular occlusion” OR “vascular exclusion” OR “vascular clamping” OR “vessel clamping” OR “vessel exclusion” OR “vein occlusion” OR “vein clamping” OR “vein exclusion” OR “portal occlusion” OR “portal clamping” OR “portal exclusion” OR “triad occlusion” OR “triad clamping” OR “triad exclusion” OR “Hanging Maneuver” OR “pedicle occlusion” OR “pedicle clamping” OR “pedicle exclusion” OR “occlusion of inflow” OR “occlusion of flow” OR “occlusion of vascular” OR “occlusion of vein” OR “occlusion of vessel” OR “occlusion of the hepatic pedicle” OR “occlusion of the total hepatic pedicle” OR “occlusion of hepatic inflow” OR “occlusion of hepatic flow” OR “occlusion of hepatic vascular” OR “occlusion of hepatic vein” OR “occlusion of hepatic vessel” OR “clamping of inflow” OR “clamping of flow” OR “clamping of vascular” OR “clamping of vein” OR “clamping of vessel” OR “clamping of the hepatic pedicle” OR “clamping of the total hepatic pedicle” OR “clamping of hepatic inflow” OR “clamping of hepatic flow” OR “clamping of hepatic vascular” OR “clamping of hepatic vein” OR “clamping of hepatic vessel” OR “exclusion of inflow” OR “exclusion of flow” OR “exclusion of vascular” OR “exclusion of vein” OR “exclusion of vessel” OR “exclusion of the hepatic pedicle” OR “exclusion of the total hepatic pedicle” OR “exclusion of hepatic inflow” OR “exclusion of hepatic flow” OR “exclusion of hepatic vascular” OR “exclusion of hepatic vein” OR “exclusion of hepatic vessel” OR “occlusion of the inflow” OR “occlusion of the flow” OR “occlusion of the vascular” OR “occlusion of the vein” OR “occlusion of the vessel” OR “occlusion of the hepatic inflow” OR “occlusion of the hepatic flow” OR “occlusion of the hepatic vascular” OR “occlusion of the hepatic vein” OR “occlusion of the hepatic vessel” OR “clamping of the inflow” OR “clamping of the flow” OR “clamping of the vascular” OR “clamping of the vein” OR “clamping of the vessel” OR “clamping of the hepatic inflow” OR “clamping of the hepatic flow” OR “clamping of the hepatic vascular” OR “clamping of the hepatic vein” OR “clamping of the hepatic vessel” OR “exclusion of the inflow” OR “exclusion of the flow” OR “exclusion of the vascular” OR “exclusion of the vein” OR “exclusion of the vessel” OR “exclusion of the hepatic inflow” OR “exclusion of the hepatic flow” OR “exclusion of the hepatic vascular” OR “exclusion of the hepatic vein” OR “exclusion of the hepatic vessel”) AND (Recurrence OR Recurrences OR Recrudescence OR Recrudescences OR Relapse OR Relapses OR “disease-free survival” OR “disease free survival”) AND (“liver” OR “hepatic”)

**Supplemental Figures:**

**Supplemental Figure 1 (A)** Forest plot showing one-year recurrence of hepatic malignant lesions after hepatectomy in patients with primary and metastatic tumors. **(B)** Forest plot showing three-year recurrence of hepatic malignant lesions after hepatectomy in patients with primary and metastatic tumors. **(C)** Forest plot showing five-year recurrence of hepatic malignant lesions after hepatectomy in patients with primary and metastatic tumors.

**Supplemental Figure 2 (A)** Forest plot showing one-year survival after hepatectomy in patients with primary and metastatic tumors. **(B)** Forest plot showing three-year survival after hepatectomy in patients with primary and metastatic tumors. **(C)** Forest plot showing five-year survival after hepatectomy in patients with primary and metastatic tumors.
