# Supplementary figures and images for: Meta-analysis of the effect of the pringle maneuver on long-term oncological outcomes following liver resection
Source: Sci Rep. 2021 Feb 8;11:3279. doi: 10.1038/s41598-021-82291-4 (PMC7870962; doi:10.1038/s41598-021-82291-4)

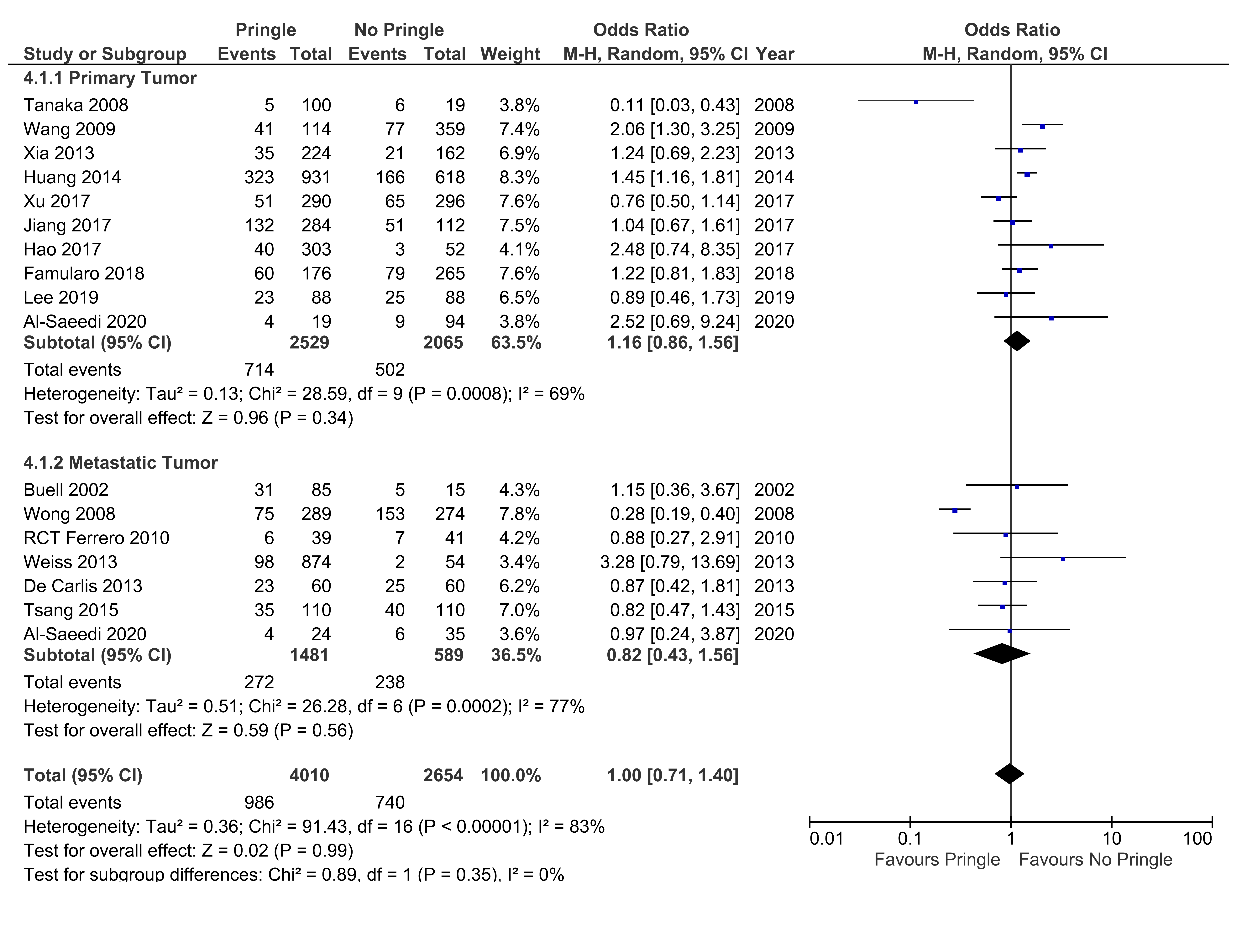

Supplement: Supplementary file 2 — Supplementary Information 2. [file 41598_2021_82291_MOESM2_ESM.tiff]

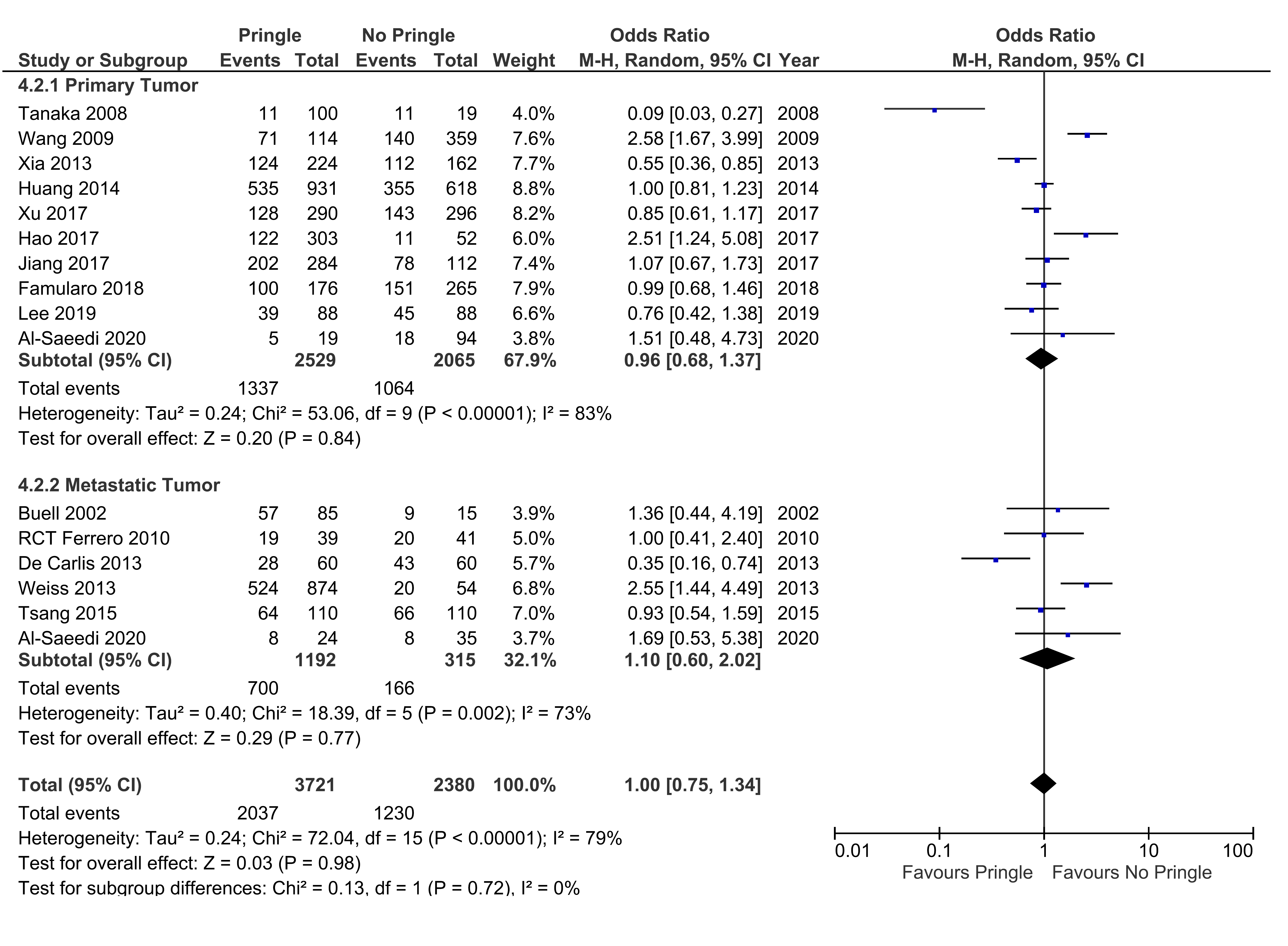

Supplement: Supplementary file 3 — Supplementary Information 3. [file 41598_2021_82291_MOESM3_ESM.tiff]

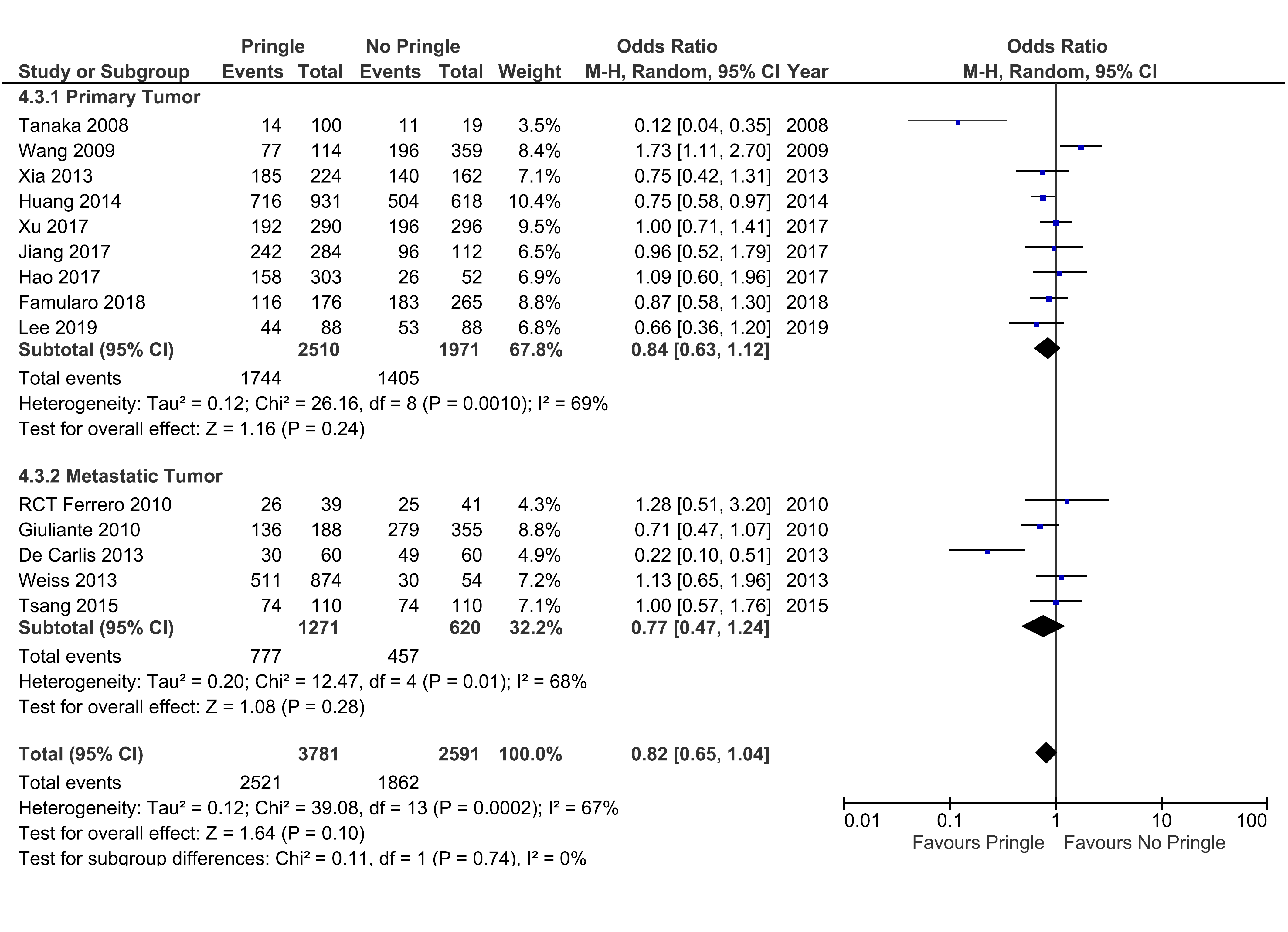

Supplement: Supplementary file 4 — Supplementary Information 4. [file 41598_2021_82291_MOESM4_ESM.tiff]

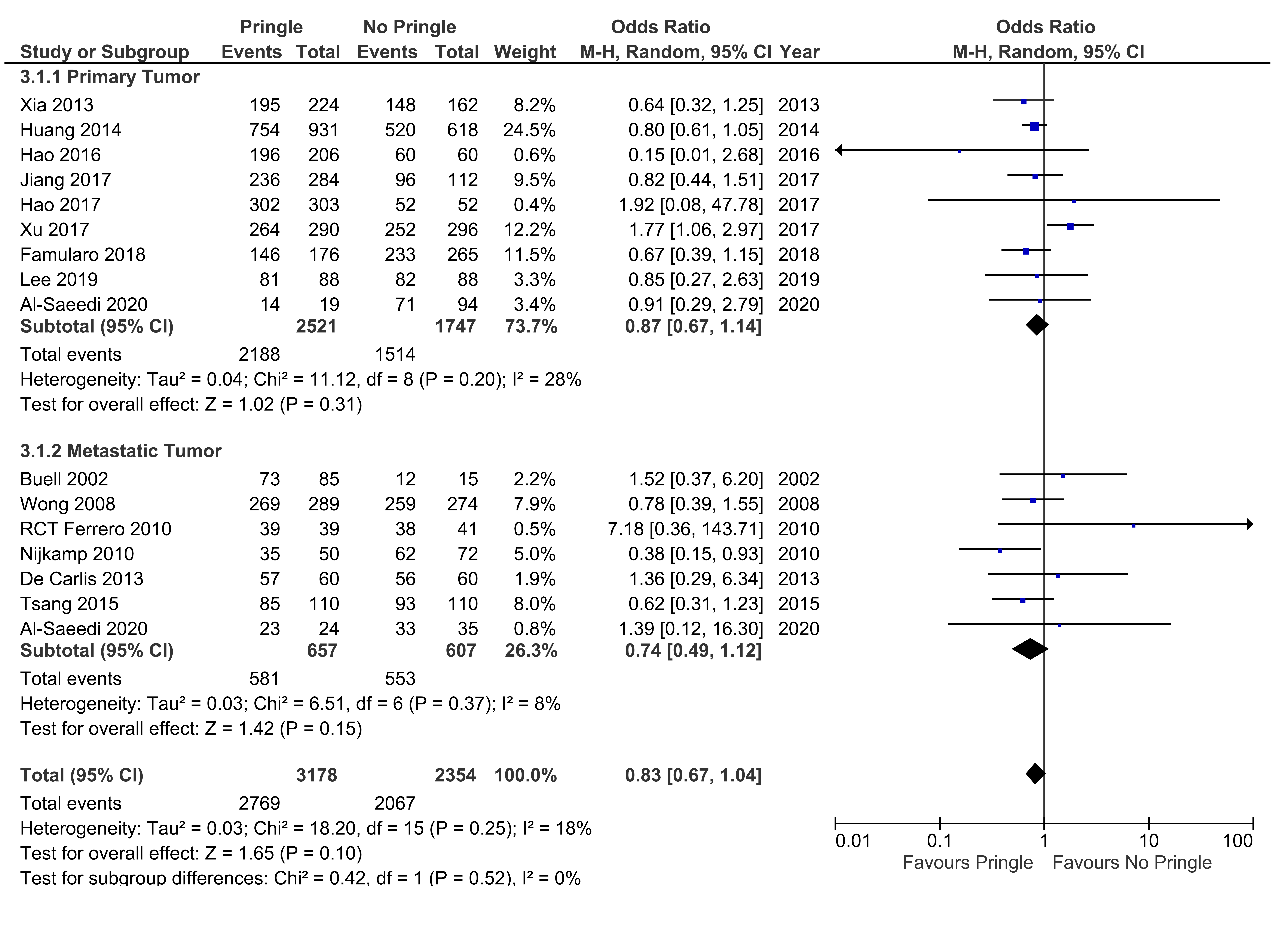

Supplement: Supplementary file 5 — Supplementary Information 5. [file 41598_2021_82291_MOESM5_ESM.tiff]

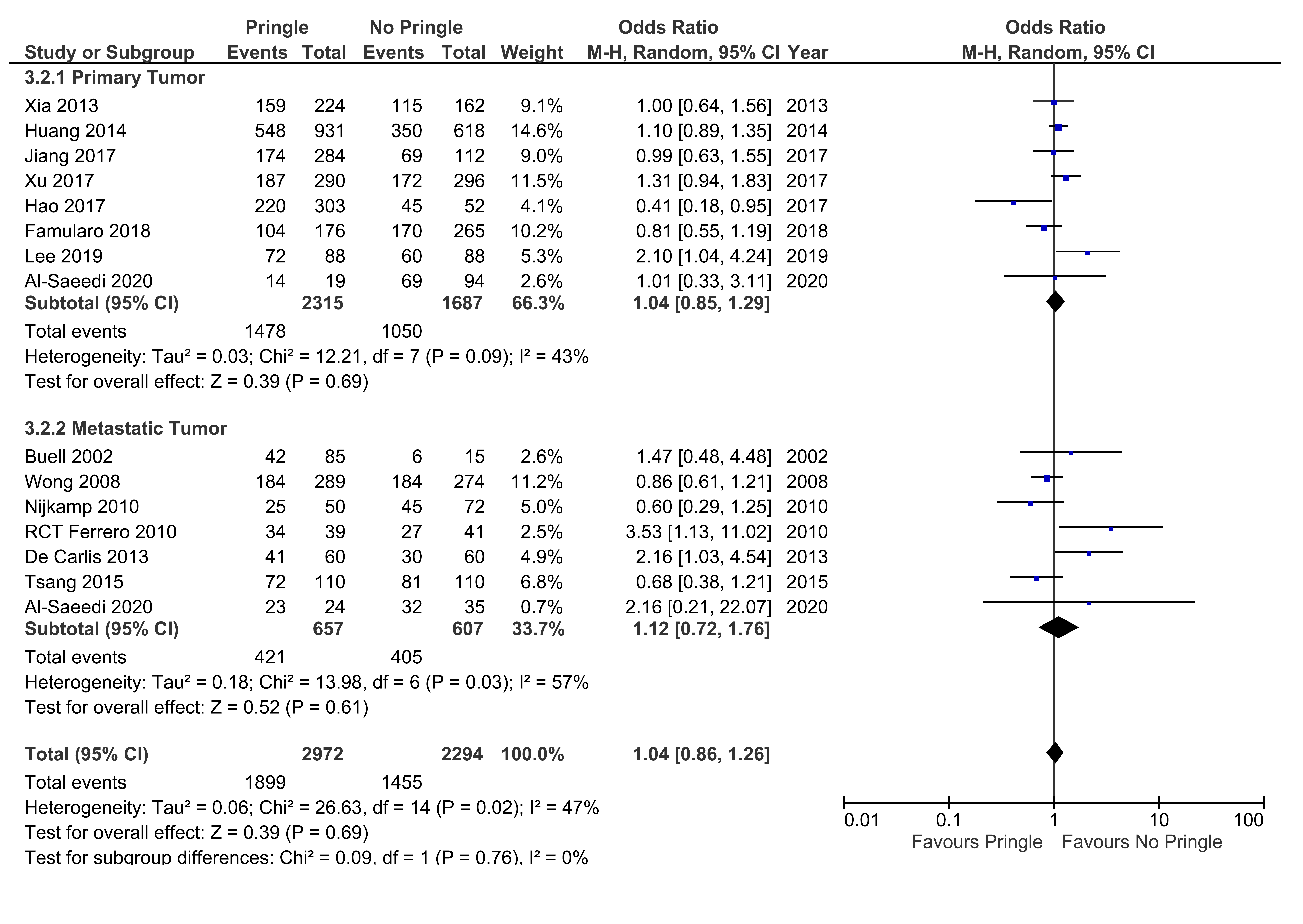

Supplement: Supplementary file 6 — Supplementary Information 6. [file 41598_2021_82291_MOESM6_ESM.tiff]

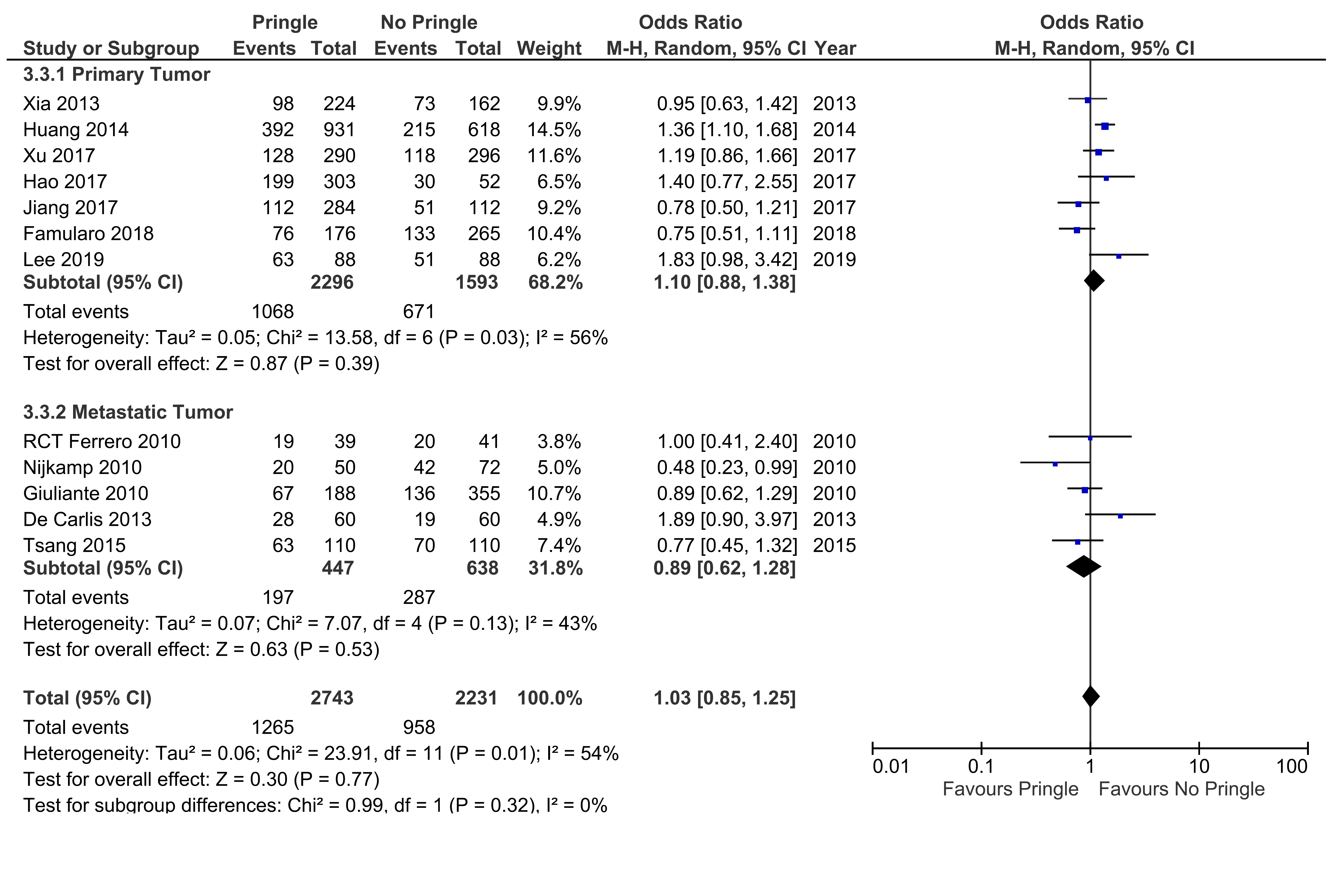

Supplement: Supplementary file 7 — Supplementary Information 7. [file 41598_2021_82291_MOESM7_ESM.tiff]
